# Engineering the glioblastoma microenvironment with bioactive nanoparticles for effective immunotherapy

**DOI:** 10.1039/d3ra01153d

**Published:** 2023-10-27

**Authors:** Ryan Blanchard, Isaac Adjei

**Affiliations:** a Department of Biomedical Engineering, Texas A&M University TX USA adjeii@tamu.edu

## Abstract

While immunotherapies have revolutionized treatment for other cancers, glioblastoma multiforme (GBM) patients have not shown similar positive responses. The limited response to immunotherapies is partly due to the unique challenges associated with the GBM tumor microenvironment (TME), which promotes resistance to immunotherapies, causing many promising therapies to fail. There is, therefore, an urgent need to develop strategies that make the TME immune permissive to promote treatment efficacy. Bioactive nano-delivery systems, in which the nanoparticle, due to its chemical composition, provides the pharmacological function, have recently emerged as an encouraging option for enhancing the efficacy of immunotherapeutics. These systems are designed to overcome immunosuppressive mechanisms in the TME to improve the efficacy of a therapy. This review will discuss different aspects of the TME and how they impede therapy success. Then, we will summarize recent developments in TME-modifying nanotherapeutics and the *in vitro* models utilized to facilitate these advances.

## Abstract 2

This review highlights recent advances in nanotherapeutics altering the tumor microenvironment of glioblastoma multiforme (GBM) to overcome immunosuppression.

## Graphical abstract

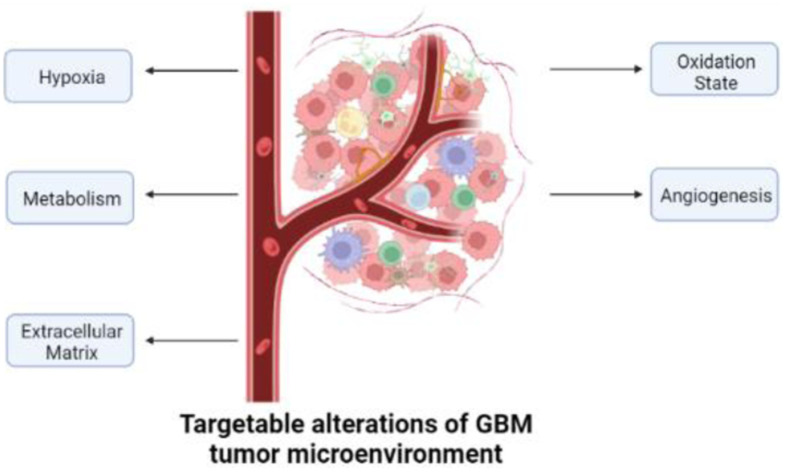

## 1. Introduction

Glioblastoma multiforme (GBM) is the most common and severe type of primary brain cancer, with a country-dependent incidence rate of 0.6–5 per 100 000 individuals.^1,2^ GBM develops spontaneously in glial cells, with the highest incidence in persons aged 45–65.^3^ Even with aggressive treatments comprising surgical resection, radiotherapy, and chemotherapy (temozolomide), the median survival time after diagnosis is only 14.6 months.^4^ While immunotherapy has improved survival for patients with different cancers, particularly liquid cancers, GBM patients have not benefited from these therapeutic advances, and outcomes have remained stagnant for almost twenty years. Unfortunately, novel immunotherapies for GBM that are successful preclinically have not progressed beyond stage 3 clinical trials.^5^

Several factors account for the failure of most GBM therapeutics. GBM tumors are genetically heterogeneous, making the design of targeted therapies challenging.^6,7^ Moreover, GBM tumors mold their microenvironment to support their growth by altering the phenotype of stromal cells, modulating blood vessel growth, and modifying the composition of the extracellular matrix (ECM). The altered GBM tumor microenvironment (TME) promotes resistance to immunotherapies by modulating hypoxia, oxidation state, and metabolism.^8,9^ In addition to TME-induced immune evasion, GBM cell niches resistant to immunotherapies arise during treatment that promote recurrence.^10^ These cell niches, often composed of self-renewing GBM stem-like cells (GSC), are maintained by the TME, which stimulates survival mechanisms.^10^ Lastly, the blood–brain barrier (BBB), which shields the brain from hazardous substances, creates a physical barrier that prevents the transport of therapeutics into the brain, hampering their success. The BBB and strategies for bypassing it are discussed in these excellent reviews and not covered in detail here.^11–13^

Nanoparticles (NPs), which can be fine-tuned as precision drug carriers or be bioactive, are promising tools to overcome TME-induced evasion or resistance to immunotherapy. NPs present unique benefits for GBM treatment due to their ability to cross the BBB. By modifying the surface of the NP, therapies can maximize BBB transcytosis and GBM targeting.^12–14^ While the tunability of nanoparticles has increased their potential for delivering therapeutics into GBMs to improve efficacy, a new class of nanoparticles that act as therapeutics themselves due to their chemical properties offers novel avenues for treatment. These NPs, responsive to biochemical cues in the TME, allow for conventional immunotherapies to be more effective. While numerous reviews discuss immunotherapy for GBM and NP-based GBM therapy, none currently examine this emerging class of NPs and their potential to alter the GBM TME. Therefore, this review will discuss the GBM TME, its role in disease progression and resistance to immunotherapy, and nanotherapeutic platforms designed to overcome these mechanisms and improve therapeutic efficacy.

## 2. The glioblastoma microenvironment

**Fig. 1:**
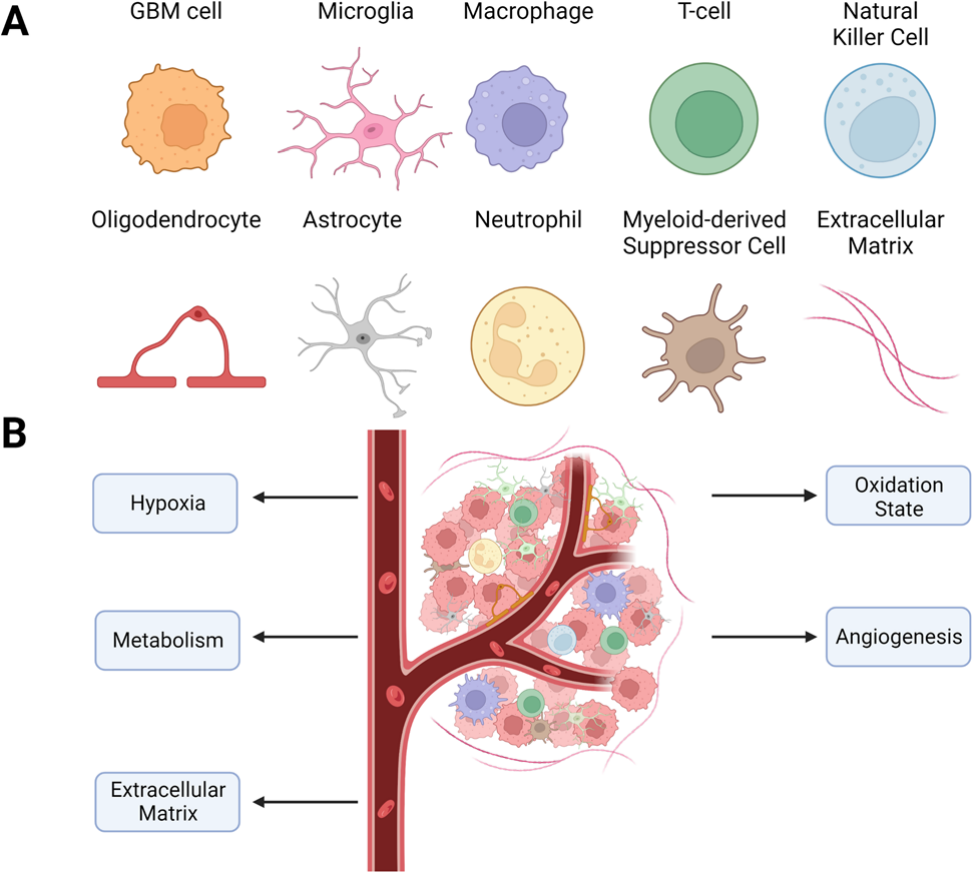
Cellular composition of the GBM TME. (A) Types of cells found within the TME. The TME is highly heterogeneous, containing various stromal and immune cells that enable cancer growth. (B) Schematic of the TME with targetable alterations currently being investigated to improve GBM therapy.

The GBM tumors are a complex mix of non-malignant cells, matrix, and molecules in addition to the cancer cells. The cancer cells co-opt these other microenvironment components to promote survival, immune evasion, and growth (Fig. 1). Changes in the microenvironment, such as oxygen level, acidity, and oxidation state, shape the phenotype and metabolism of malignant and non-malignant cells. However, unlike genomic changes to GBM cells, which are heterogeneous and vary within and between tumors, alterations to the TME are generally consistent across tumors. This consistency of changes in the TME across tumors makes it an exciting target for therapeutic development.

### 2.1 Cellular composition

#### 2.1.1 GBM cells

GBM cells are derived from astrocytes, the glial cells that maintain a supportive environment for neuronal signaling in the central nervous system (CNS). GBM develops through genetic mutations, including those involved in cell cycle arrest, such as p53, RTK/RAS/PI3K, retinoblastoma (RB), and isocitrate dehydrogenase (IDH1) that promote uncontrolled GBM cell proliferation.^15,16^ Other mutations promote the remodeling of their microenvironment, co-opting the neighboring neurons, glial cells, cancer-associated fibroblasts (CAF), endothelial cells, and immune cells. Specifically, changes that increase GBM cells' secretion of cytokines, such as transforming growth factor-β (TGF-β) and interleukin-10 (IL-10), promote immunosuppressive cell recruitment and cancer progression.^17^ By suppressing the activity of immune cells, GBM cells can proliferate without checks on their growth.

#### 2.1.2 Stromal cells

GBM-associated stromal cells (GASC) and glial cells, which can be divided primarily into astrocytes, oligodendrocytes (Section 2.1.2.1), and microglia (Section 2.1.3), contribute to the formation and modification of the TME (Section 2.1.2.2).^18^

##### 2.1.2.1 Glioblastoma-associated stromal cells (GASC)

The presence of fibroblast-like cells in GBM tumors is a highly disputed question due to the lack of fibroblasts in healthy brain tissue. However, recent studies have identified GASC populations with myofibroblast properties that drive ECM remodeling and angiogenesis.^19–21^ These cells are associated with tumor progression and stemness, driving negative patient outcomes.^22^ One GASC population, which is mesenchymal stem cell-like, constructs a pro-invasive matrix through force-mediated ECM remodeling.^21^ Another GASC phenotype in the surgical margins of GBM tumors promotes tumor and endothelium development by stimulating angiogenesis.^19^ GASCs also promote the expansion of the GSC populations through the secretion of hepatocyte growth factor (HGF) and can induce M2 polarization through extra domain A fibronectin.^23^ These identified cells facilitate cell migration, drug resistance, and angiogenesis through the secretion of paracrine factors such as TGF-β and vascular endothelial growth factor (VEGF).^24–27^

##### 2.1.2.2 Glial cells

Astrocytes maintain the homeostasis of neuron metabolism and mediate neuronal communication with microglia, monocytes, and T cells.^28,29^ While the pathophysiology of astrocytes within GBM is not well-defined, recent work shows that reactive astrocytes secrete the anti-inflammatory cytokines TGF-β, IL-10 and granulocyte colony-stimulating factor (G-CSF) that also have immune suppressive properties.^30–32^

Oligodendrocytes are rare within the TME but increase in number at the tumor border.^33,34^ There, oligodendrocyte progenitor cells induce stemness and chemoresistance.^34^

##### 2.1.2.3 Neurons

Neurons stimulate the proliferation of GBM cells through the secretion of neuroligin-3, which activates the PI3K-mTOR pathway.^35^ The PI3K-mTOR pathway is continuously stimulated in cancer cells, which promotes tumorigenesis and therapy resistance.^36^ In addition, neuronal signaling can activate calcium signaling in populations of GBM cells, driving microtube formation and invasion.^37^

#### 2.1.3 Myeloid immune cells

The largest population of immune cells in the GBM tumor are the myeloid-derived cells, including microglia cells, macrophages, myeloid-derived suppressor cells (MDSC), neutrophils, and dendritic cells (DC).^38^ Microglia are glial cells that perform resident macrophage functions in the brain and represent the largest immune cell population in the TME. While macrophages are not present in the healthy CNS, monocytes enter and differentiate into macrophages when the integrity of the BBB is impaired.^39^ The microglia and tumor-associated macrophages (TAMs) skew towards the M2 polarization state, exerting pro-tumor effects by inhibiting T cell cytotoxicity through contact and autocrine mechanisms or promoting their apoptosis by expressing the Fas ligand.^40,41^

Neutrophils accumulate in the TME and promote GBM cell proliferation and epithelial-to-mesenchymal transition behavior by expressing S100A4.^42,43^ While DCs are absent in healthy brain tissue, they migrate to the CNS after tumorigenesis to support tumor rejection.^44^ However, GBM cells inhibit DC maturation and thus suppress their antigen-presenting function while promoting their role in anergy by stimulating the release of the redox-sensitive transcription factor nuclear factor erythroid 2-related factor 2 (Nrf2).^45^ MDSCs are a heterogeneous population of myeloid cells that promote the immunosuppression of natural killer (NK) cells and cytotoxic T cells through a variety of pathways, including nitric oxide, prostaglandin E2 (PGE2), and arginase 1 (ARG1).^46^

#### 2.1.4 Lymphoid immune cells

Regulatory T cells (*T*_reg_), characterized by their expression FOXP3, induce tolerance in cytotoxic T cells by expressing PD-1 and CTLA-4.^47^ In addition, constant activation and exposure to stimuli such as reactive oxygen species (ROS) leads to telomere shortening in cytotoxic T cells, resulting in their senescence.^48^ NK cells are innate cytotoxic immune cells that can target cells without MHCI, allowing them to target cells that escape cytotoxic T cells.^49^ TGF-β causes a decrease in the expression of NKG2D, an activating receptor expressed on NK and cytotoxic T cells that activates an antitumor response, reducing cytotoxic activity in GBM patients.^50^ While T cells are often mostly dysfunctional in the GBM TME, NK cells have shown a capacity for cytotoxicity.^51^ While NK cells are relatively uncommon in the TME, their behavior has made them an area of interest for new GBM immunotherapies.^52^

### 2.2 Vascular changes and hypoxia in the TME

The high rates of cellular growth and increased VEGF expression result in a vascular network that is tortuous and disorganized, with larger diameters than normal blood vessels.^53,54^ Increased permeability and disorganization lead to unequal blood flow in the tumor, which causes the formation of hypoxic and necrotic zones.^55^

Hypoxia is a hallmark of GBM and leads to increased invasiveness and aggressiveness.^55,56^ Cells in hypoxic zones form hypercellular pseudopalisades, which promote migration and aggressiveness.^57^ Hypoxia increases the expression of the hypoxia-inducible factor (HIF-1α), which controls angiogenic, transcriptional, and metabolic pathways.^58,59^ HIF-1α induces the expression of matrix metalloproteinases (MMP) that contribute to invasion by remodeling the ECM.^60^ Hypoxia increases the expression of multidrug resistance-associated protein 1 (MRP-1), resulting in therapy resistance by pumping drugs out of the cells.^61^ Hypoxia also increases autophagy or self-digestion, a survival mechanism GBM cells use to avoid apoptosis by activating the PI3K/MAPK pathway.^62^ Autophagy leads to resistance to TMZ and radiation therapy.^63^

### 2.3 Metabolic changes in the TME

#### 2.3.1 GBM cell metabolism

Cancer cells alter their metabolism to support survival and proliferation and produce metabolites that promote immune evasion. While normal cells rely primarily on oxidative phosphorylation for ATP production, cancerous cells shift to aerobic glycolysis, diverting pyruvate to extra-mitochondria lactate production.^64^ The increase in lactate concentrations, particularly in hypoxic regions, can act as an oxidative substrate for cancer cells.^65,66^ Glutamine uptake is increased in GBM cells and provides building blocks for synthesizing nucleotides, other amino acids, and fatty acids by entering the tricarboxylic acid (TCA) cycle.^67^ While aerobic glycolysis boosts ATP production, the main benefit of the metabolic switch for GBM cells is the added production of intermediates to build nucleotides, lipids, proteins, and fatty acids for tumor expansion.^68^ This switch to aerobic glycolysis is also associated with increased ROS levels that facilitate cancer cell adaptation to hypoxia by ROS-mediated HIF-1α stabilization.^69^ HIF-1α, in turn, regulates glycolytic genes such as GLUT1, HK2, PFKFB3, and PGK1, which drives glycolysis in hypoxic regions.^70^

Lactate transported out of tumor cells lowers the extracellular pH from approximately 7.4 on the leading edges to approximately 6.0 in the central necrotic zones of the tumor.^71,72^ Cells re-establish intracellular pH levels by importing additional bicarbonate ions, facilitated by the hypoxia-induced downstream protein carbonic anhydrase IX (CA-IX).^73^ Acidification of the tumor prompts significantly higher levels of immunosuppression and GSC migration and proliferation.^74–76^

Molecular building blocks such as amino acids and nucleosides also have increased concentrations within the TME, contributing to greater aggression, invasion, and immunosuppression. Adenosine, a potent immunosuppressant produced in large quantities in hypoxic conditions by GBM cells and exported into the extracellular space, increases cancer aggressiveness and dampens the immune response through A2A receptors (A2AR) on immune cells.^77–79^ Arginine promotes tumorigenesis and angiogenesis^80^ and is provided by external sources since the urea cycle, the primary source of arginine, is inhibited in GBM cells through the silencing of argininosuccinate synthetase 1.^81^

#### 2.3.2 Immune cell metabolism

The tumor microenvironment alters the metabolism of immune cells. TAMs, the most abundant immune cell population in the TME, preferentially accumulate in hypoxic regions, increase HIF-1α expression, and promote metabolic switching to glycolysis.^82^ This leads to increased ROS and reactive nitrogen species by TAMs, which in turn causes an increase in the expression of the immune checkpoint protein programmed cell death ligand 1 (PD-L1) and M2 polarization.^83,84^ The high metabolic activity of MDSCs depletes essential amino acids and generates immunosuppressive amino acid metabolites that suppress T cells.^85^ Increased expression of indoleamine 2,3-dioxygenase (IDO) in MDSCs supports the production of kynurenines from tryptophan, while increased expression of ARG1 deplete l-arginine and sequest l-cysteine.^67,86^ ARG1, NOS2, and NADPH oxidase lead to ROS and reactive nitrogen species (RNS) generation from MDSCs.^85^

T cells rely primarily on fatty acid oxidation and oxidative phosphorylation in their quiescent state.^87^ When activated, the energy needs increase, which is met by enhanced requirements of glucose, glutamine, and l-arginine.^88^ The TME is poor in glucose and amino acids, which hinders proliferation and effector functions such as TCR signaling and inflammatory cytokine secretion.^67,89^ On the other hand, *T*_reg_ cells rely primarily on fatty acid oxidation and can proliferate more easily within the TME. Naïve T cells in the TME will preferentially differentiate into *T*_reg_ cells instead of cytotoxic T cells due to AMP-activated protein kinase (AMPK1), which senses and is expressed in environments of nutrient deficiency.^90^

TGF-β inhibits the IL-2-induced metabolism of NK cells, decreasing oxidative phosphorylation.^91^ Excess adenosine in the TME stimulates NK cells through A2AR, decreasing their cytotoxicity.^92^ In addition, lactic acid blocks IFN-γ expression in cytotoxic T cells and NK cells by inhibiting the nuclear factor of activated T cells (NFAT).^93^ Like T cells, depletion of glucose and amino acids impairs NK cell function and proliferation.^94,95^

#### 2.3.3 Oxidation state

A byproduct of increased metabolism is elevated ROS generation by cancer cells. Usually, ROS, such as superoxide (O_2_˙^−^), which can be converted to hydrogen peroxide (H_2_O_2_) or hydroxyl radical (OH˙) by superoxide dismutase (SOD), are primarily produced through oxidative phosphorylation.^96^ As the most stable and permeable ROS, H_2_O_2_ is vital for signaling transcription factors such as HIF-1, NOTCH, nuclear factor kappa B (NF-κB), and P-53.^97^ In GBM, increased levels of superoxide dismutase (SOD) lead to a greater conversion rate of the unstable O_2_˙^−^ into H_2_O_2_, which, together with an elevated basal metabolic rate, causes the accumulation of H_2_O_2_ in the TME.^98^ This increase in ROS in the TME upregulates the expression of transcription factors such as Forkhead box protein O1 (FOXO) and Activating Protein-1 (AP-1) and the activation of protein kinases, stimulating proliferation and aggression.^99,100^ ROS in the CNS, which is particularly susceptible to oxidative damage, leads to genetic instability by inhibiting DNA repair, cell damage through reactions with DNA, RNA, and lipids, and an increase in proliferation, angiogenesis, and migration in the tumor.^96^ In areas experiencing hypoxia, ROS concentration is exceptionally high through inhibition of the electron transport chain in mitochondria.^101^

Cancer cells increase the production of antioxidants such as glutathione (GSH) and catalase to prevent ROS-induced apoptosis.^98^ Catalase is an enzyme that catalyzes the reaction of hydrogen peroxide into oxygen and water, while GSH is oxidized glutathione disulfide (GSSG), which reduces hydrogen peroxide to oxygen and water. In the TME, these antioxidants induce resistance to the application of therapies. GSH elevates the expression of multidrug resistance protein, and accumulation of GSSG has adverse effects on metabolic regulation and cellular integrity.^102,103^ While overexpression of catalase reduces levels of H_2_O_2_, it also leads to increased proliferation, resistance to temozolomide and radiotherapy, and the formation of neurospheres, a feature of GSCs.^104^

### 2.4 Changes to the ECM in the TME

**Fig. 2:**
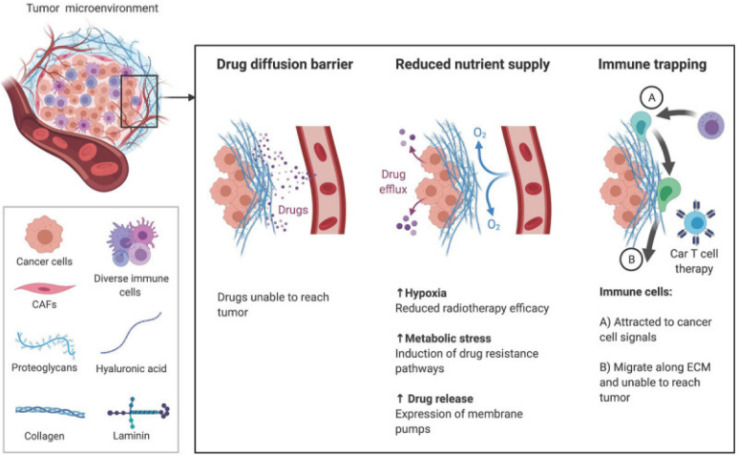
The TME ECM impairs therapy through increased stiffness and density. Increased pressure prevents the transport of drugs and nutrients to tumor and movement of immune cells toward chemokine signals [reproduced from ref. 188 with permission from AJCR, ©2021].

The brain ECM contains a uniquely flexible matrix that structures the parenchyma and guides signaling pathways that regulate cellular functions such as differentiation and migration.^105^ The main components of the ECM are glycosaminoglycans (GAG) and glycoproteins such as laminins, fibronectin, and tenascins.^106^ The brain contains less fibrous proteins, such as collagen, than other tissues.^107^ In GBM, the ECM increases in stiffness and density, leading to a rise in interstitial pressure.^108^ These changes in the ECM reduce nutrient and oxygen penetration and access to the cancer cells, increasing hypoxia and metabolic stress. The effectiveness of drugs to penetrate the tumor is also reduced, minimizing their efficacy (Fig. 2).^109^

The GAG hyaluronic acid (HA) is vital in tumor progression. The molecular weight of HA determines if it inhibits or enhances tumor progression. Low molecular weight HA (<250 kDa) triggers the G1 phase to promote proliferation, while high molecular weight HA (>1000 kDa) locks cells in the G1 phase.^110,111^ The expression of the adhesion receptor CD44, which binds to HA, is associated with increased GBM aggressiveness, proliferation, chemoresistance, and stemness.^112^ Chondroitin sulfate proteoglycan-4 (CSPG-4) is another GAG upregulated in GBM.^113^ Tumors expressing CSPG-4 have worse outcomes, as the GAG encourages migration by promoting the expression of β-integrins and growth factors.^114^ Increased collagen in the TME, which increases its stiffness, leads to immune trapping, where migration of immune cells such as T cells is physically inhibited.^115^

A variety of glycoproteins are also upregulated in GBM. Tenascin-C (TN-C) is expressed almost exclusively in GBM ECM *versus* healthy brain ECM, promotes cell migration and angiogenesis, and mediates changes in ECM constituents.^116,117^ Fibronectin increases the adhesion, proliferation, and resistance of GSCs.^118^ Fibulin-3, a glycoprotein only found in cancerous brain tissue, promotes tumor progression through the Notch and NF-κB signaling pathways, enhancing the viability of tumor-initiating cells.^119,120^

One of the most critical components related to the ECM is matrix metalloproteinases (MMP), enzymes that degrade and remodel the ECM. MMP-2 and MMP-9 are upregulated in GBM, with MMP-9 expression changing from undetectable in the healthy brain to significantly expressed in GBM.^121,122^ Both molecules promote cancer malignancy. MMP-2 promotes the transition to an invasive, undifferentiated phenotype, alters the metabolism, and inhibits apoptosis.^123,124^ Similarly, MMP-9 is essential in instigating GBM invasion.^125^

## 3. *In vitro* models to study TME

**Fig. 3:**
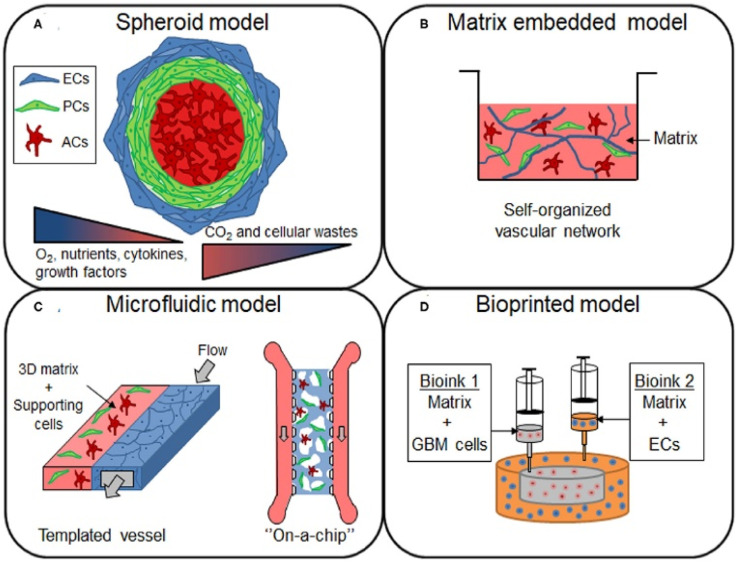
*In vitro* models for studying the GBM TME. (A) Spheroid models are constructed by using cell–cell interactions to form cell aggregates of one or more cell types in a culture. These models are ideal for the study of hypoxia and nutrient gradients, as spheroids replicate the necrotic cores found in human GBM tumors. (B) Cells or spheroids can be embedded in a matrix containing natural or synthetic materials that replicate the ECM. (C) Microfluidic models, which can be customized for the application, are gaining popularity. These models are ideal for dynamic models, where the effects of flow on the TME can be observed by flowing media across fabricated vessels coated with matrix or cells. (D) Bioinks are used to 3D print models containing various types of cells. This can be utilized to easily construct a custom scaffold that better reflects the structure and function of a GBM tumor. Models can be constructed from commercially available cell lines or patient-derived cells using various model approaches depending on the application [reproduced from ref. 189 with permission from Frontiers, ©2021].

*In vitro* models are used in conjunction with *in vivo* models to understand the TME and its effects on therapeutic efficacy better.^126^ However, standard two-dimensional cell culture models fail to reproduce the unique conditions in the TME, limiting their applications in studying the TME and evaluating new cancer therapeutics.^127^ In 2D models, cell–ECM and cell–cell interactions are superseded by cell-tissue culture plastic interactions.^128^ Therefore, three-dimensional tumor models that recapitulate dysfunctional aspects of the TME, such as hypoxia gradients and cell-ECM interactions, are necessary (Fig. 3).^129^

Spheroids, which are 3D aggregations of cells often used as tumor models, are formed using various techniques, including bioprinting and aggregation on a low attachment surface such as agarose in static or dynamic conditions.^130^ Once generated, spheroids can be cultured in a scaffold or a scaffold-free environment. Scaffolds, or matrices, allow researchers to mimic the ECM of the TME, replicating some of the organizational structure *in vitro*.^131^ For GBM research, the most common materials are natural biomaterials such as Matrigel, HA, collagen, and decellularized ECM or synthetic polymers such as poly(ethylene glycol) (PEG) and poly(urethane).^132,133^ A critical variable in constructing a model is cell choice. The most common choice is a glioblastoma cell line, such as human U87 or mouse GL261. However, these cell lines lack the heterogeneity in GBM tumors; therefore, many researchers utilize patient-derived organoids.^134^ By collecting patient-derived GSCs for organoid generation, models can reflect the structure of the tumor rather than that of the normal brain.^135^ The last primary consideration for organoid models is co-culturing cancer cells with non-malignant tumor cells. For example, organoids constructed of GSCs can be augmented with human cerebral organoids to study invasion into the brain.^136,137^ These cerebral co-culture models are derived from mouse embryonic stem cells or genetically engineered human embryonic stem cells.^138^ Additionally, through rapid 3D bioprinting combining GSCs with astrocytes, neural precursor cells, and macrophages in an HA scaffold, researchers were able to recapture cellular interactions and immune functions.^139^

Although organoids are currently popular for studying the TME, microfluidic lab-on-a-chip models have risen as alternatives. These devices, fabricated mostly from poly (dimethylsiloxane) (PDMS) using soft lithography, offer flexibility in design choices and model complexity that are not available in organoid models. After fabrication, vessels within the chip can be coated with a protein that promotes cell attachment, such as fibronectin, and a matrix to model the TME. Lab-on-a-chip models are ideal for many situations, particularly studies involving fluid flow, cell migration, and drug discovery.^140^ Like organoids, cell choice and scaffold material are critical decisions for the design of lab-on-a-chip models. Most researchers utilize a human GBM cell line such as U87MG, with other studies also constructing co-cultures with HUVECs, astrocytes, or hCMEC/d3, a brain endothelial cell line.^141^

## 4. Techniques to engineer the TME with nanotechnology

The changes in the GBM TME described above have been under investigation with the goal of developing more effective therapeutics. Recently, interest has increased in applying these advances to developing nanotherapeutic platforms targeting specific TME parts.

### 4.1 Hypoxia

**Fig. 4:**
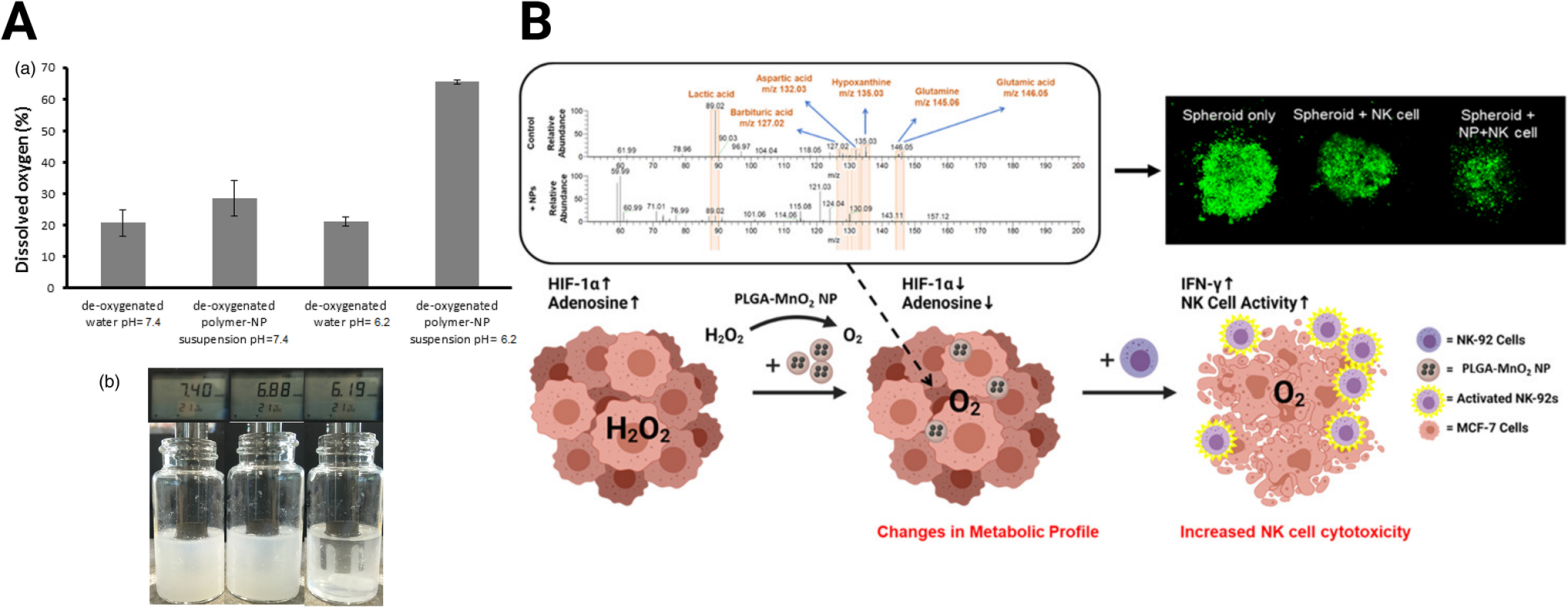
Oxide-based NPs for hypoxia reduction. (A) Dissolved oxygen generated by CaO_2_ NPs with changes in pH [reproduced from ref. 142 with permission from Elsevier, ©2017]. (B) MnO_2_ NPs skew TME from immunosuppression and improve adoptive NK cell therapy [reproduced from ref. 144 with permission from American Chemical Society, ©2021].

Hypoxia is a central focus of TME-targeting therapies. The most direct strategy developed to reduce hypoxia levels utilizes metal oxide NPs. These NPs react with various molecules in the TME to produce oxygen. For example, calcium peroxide (CaO_2_) NPs form oxygen in the presence of water (eqn (1A)). However, because of the ubiquity of water, this reaction is non-specific, and the NPs react immediately after administration. Coating CaO_2_ NPs with a pH-sensitive polymer for oxygen production in the acidic environment in pancreatic cancer addresses this drawback.^142^ These NPs generate oxygen in the acidic tumor but produce minimal oxygen at the normal physiological pH (pH = ∼7.4) in healthy tissues (Fig. 4A). While CaO_2_ NPs have been demonstrated in some tumors, their effectiveness has not been tested in GBM.

1A 2CaO_2_ + 2H_2_O → Ca(OH)_2_ + O_2_

1B MnO_2_ + 2H_2_O_2_ → Mn^2+^ + 2H_2_O + 2O_2_

Eqn (1A) and (1B): chemical reactions of oxygen-generating NPs. (A) CaO_2_ NP reaction with H_2_O (B) MnO2 reaction with H_2_O_2_.

Due to the reaction's non-specificity, CaO_2_ NPs are utilized less than manganese oxide (MnO_2_) NPs. MnO_2_ NPs, in contrast to CaO_2_, react with hydrogen peroxide in the hypoxic tumor core to form oxygen (eqn (1B)). However, MnO_2_ NPs require stabilization, or they will aggregate.^143^ PEGylation can improve stability, but the exposed MnO_2_ NPs react too quickly to adequately shift the expression of hypoxic cancer cells in the immunosuppressive TME. This problem was addressed by encapsulating the MnO_2_ NPs in poly(lactic-*co*-glycolic acid) (MnO_2_-PLGA NP) to extend the oxygen generation and shield cells from non-specific toxicity.^144^ The authors demonstrated that delivery of the MnO_2_-PLGA NPs reduced hypoxia without an increase in cytotoxicity, skewing the expression and metabolism of breast cancer 3D spheroid models away from their immunosuppressive phenotype (Fig. 4B). Specifically, MnO_2_-PLGA NPs reduced expression of HIF-1α and presence of immunosuppressive metabolites, such as lactic acid and adenosine. These changes improved the cytotoxicity of natural killer (NK) cell immunotherapy towards the spheroids. In addition, MnO_2_ NP-based platforms have been designed specifically for GBM treatment. Liang *et al.* developed a MnO_2_ NP system to enhance sonodynamic therapy (SDT), using protoporphyrin as a sonosensitizer that utilizes generated oxygen to enhance efficacy.^145^ This therapy conjugated a high-affinity transferrin to the NP to boost BBB penetration through receptor-mediated endocytosis and decreased tumor burden in C6 GBM xenografts.

Like MnO_2_, cerium oxide (CeO_2_) leverages reacting with H_2_O_2_ for hypoxia treatment. While CeO_2_ NPs have not been utilized for GBM treatment, therapies using this technique as a sensitizer for radiotherapy or photodynamic therapy have shown promise in other cancer types.^146,147^

Another solution to reduce hypoxia is to encapsulate hypoxia-reducing drugs in NP shells. Wu *et al.* designed hypoxia-reducing silica NPs that encapsulate catalase, which degrades hydrogen peroxide to produce oxygen.^148^ These NPs were covered with aptamer-modified macrophage exosomes to improve BBB penetration and reduce uptake by the reticuloendothelial system. In addition, the silica NPs were degradable in the presence of GSH, an antioxidant elevated in the TME, boosting delivery efficiency.^149^

### 4.2 Redox

There are two primary, intertwined mechanisms that NPs can use to affect the oxidation state of the tumors. The first is the direct generation of ROS by NPs, generally through Fenton or Fenton-like reactions with metal oxide NPs. In the Fenton reaction, ferrous ions react with hydrogen peroxide to produce hydroxide radicals (eqn (2)).^150^ Taking advantage of excess H_2_O_2_ in the TME, iron oxide NPs create ROS, which are toxic to cancer cells and drive the TME towards an inflammatory phenotype.^151^ The weakly acidic TME catalyzes the Fenton reaction, effectively localizing its effect to the tumor site.^152^ Similarly, Fenton-like reactions utilize other metal ions, such as Mn^2+^, Cu^+^, and Ti^3+^, to generate hydroxyl radicals. The second method of affecting the oxidation state impacts the availability of antioxidants such as GSH and catalase.

2 Fe^2+^ + H_2_O_2_ → Fe^3+^ + ˙OH + OH^−^

Eqn (2): Fenton and Fenton-like reactions.

**Fig. 5:**
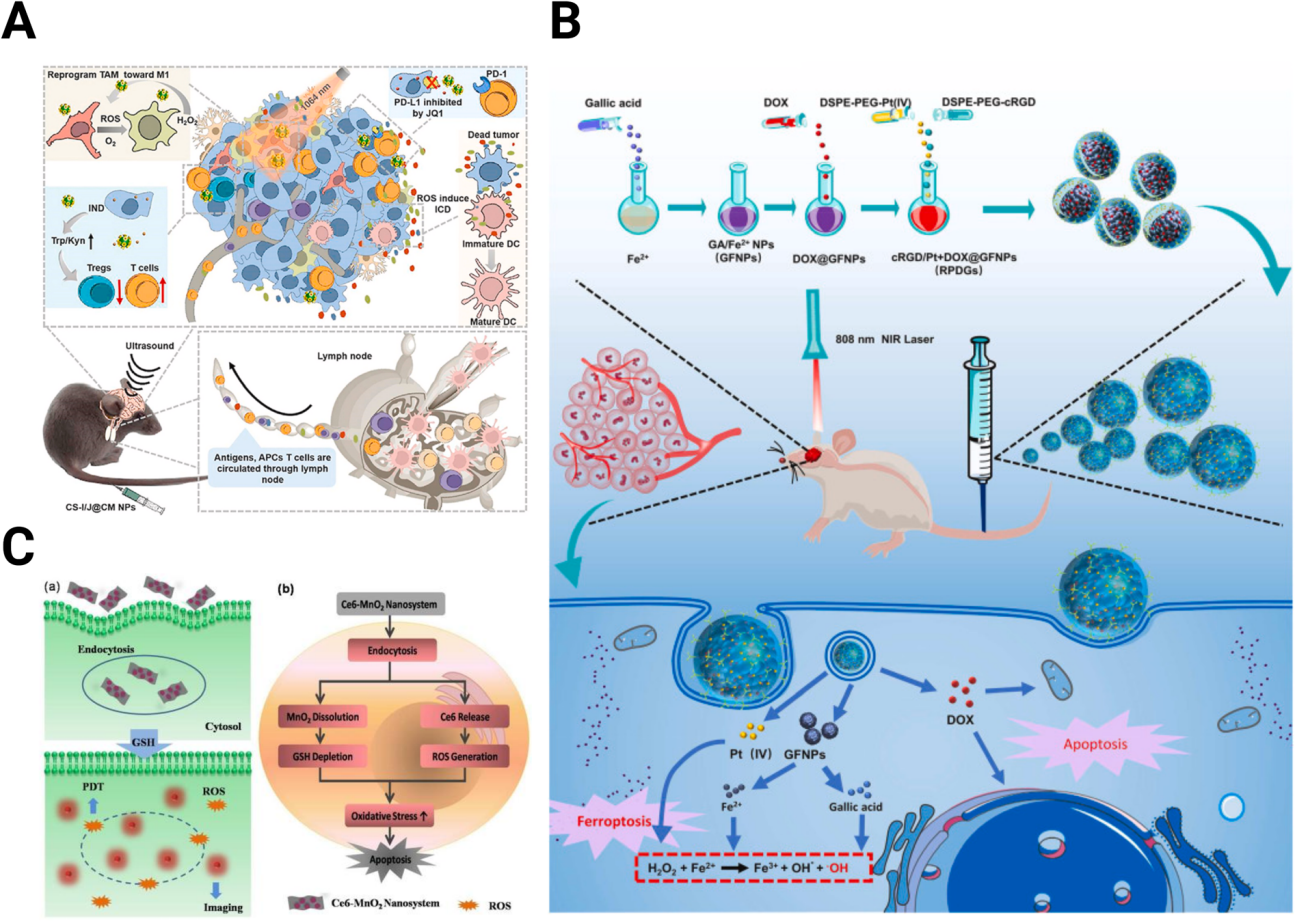
Schematics of nanomedicines utilizing oxidation state. (A) Cu_2−*x*_Se NPs for ROS production to improve checkpoint blockade therapy [reproduced from ref. 153 with permission from KeAi, ©2022]. (B) Extended Fenton reactions with gallic acid/Fe^2+^ NPs for redox disruption and effective ferroptosis [reproduced from ref. 154 with permission from Elsevier, ©2021]. (C) Ce6-modified MnO_2_ nanosheets for GSH depletion and reduced radioresistance [reproduced from ref. 158 with permission from Wiley-VGH, ©2016].

A photo-responsive NP system to produce a Fenton-like reaction was developed using ultrasmall Cu_2−*x*_Se NPs (Fig. 5A).^153^ This system, when activated with near-infrared (NIR) irradiation, generates ROS and O_2_, inducing a change in polarization of TAMs from the M2 to M1 state within the TME. Combined with the indoleamine 2,3-dioxygenase (IDO) inhibitor indoximod, these NPs improved PD-L1 blockade therapy in GBM through JQ1, which is a small molecule inhibitor of bromodomains that inhibits PD-L1 expression.^153^ Complexing Fe^2+^ with the natural anti-inflammatory agent, gallic acid could extend the Fenton reactions (Fig. 5B).^154^ These NPs strongly absorb NIR light upon exposure, which produces heat and promotes GBM cell ferroptosis, a non-apoptotic cell death dependent on intracellular iron concentration.^155,156^ Combining the complexed Fe^2+^ and gallic acid with cisplatin and a glutathione peroxidase-4 small interfering RNA (siRNA) improves the antitumor effect of the system further. Localized heating of Fe_3_O_4_ NPs stimulates the production of ROS-producing lipid peroxides, allowing for more effective cell death in prostate cancer by ferroptosis.^157^

In addition to reducing hypoxia, MnO_2_ can reduce the concentration of antioxidants, making the TME more susceptible to the generation of ROS. Fan *et al.* designed a MnO_2_-photosensitizer system for effective photodynamic therapy (Fig. 5C).^158^ MnO_2_ nanosheets protect and carry the photosensitizer chlorin e6 (Ce6) into the cell, where the MnO_2_ is reduced by GSH, depleting GSH concentrations and releasing Ce6, which generates ROS upon application of light. Yong *et al.* developed Gd-containing nanospheres with polyoxometalate-conjugated chitosan (GdW_10_@CS) for reducing hypoxia and depleting GSH to reduce radioresistance.^159^ While acting as a carrier for HIF-1α siRNA, the nanospheres trigger GSH oxidation to generate ROS through W^6+^.

### 4.3 Angiogenesis

While NP systems that target hypoxia affect new blood vessel formation, other strategies are employed to prevent angiogenesis in the TME. Most of the current research in this field focuses on synthesizing targeted and multi-functional NPs using chemotherapeutic agents such as doxorubicin (DOX) and paclitaxel and VEGF-targeting agents such as bevacizumab.^160^ For example, Lu *et al.* designed a multi-functional NP to maximize DOX efficacy by employing the anti-angiogenic drug combretastatin A4 (CA4).^161^ This tubulin-binding agent causes blood vessel regression in the TME and acts in concert with DOX and all-trans retinoic acid within an MRI-traceable iron oxide nanocube for a controlled release system (CARD-B6).^162^ The CARD-B6 platform drastically decreased blood flow within the tumor compared to free drugs and the nanoparticle platform without functionalization. Another strategy utilizes RNAi targeting VEGF using a dendrigraft poly-l-lysine gene vector NP modified by transferrin receptor for increased BBB penetration.^163^

CXCR4, the receptor for CXCL12, or SDF-1α, plays an essential role in facilitating the proliferation of GSCs and promoting angiogenesis.^164,165^ Séhédic *et al.* designed a novel lipid nanocapsule for internal radioimmunotherapy through dual rhenium-188 (^188^Re) delivery and a function-blocking antibody for CXCR4.^166^^188^Re, a beta radionuclide, acts as an internal vectorized radiotherapy. Therefore, ^188^Re is accompanied by 12G5, an antibody that blocks the function of CXCR4, to improve efficacy. The NP platform decreased tumor size and improved survival in an orthotopic U87MG mouse compared to mice treated with NP without non-specific IgG2a or saline injection. After study completion, CD31 staining of the tumor showed a significant decrease in blood vessel presence.

### 4.4 Metabolic alterations

**Fig. 6:**
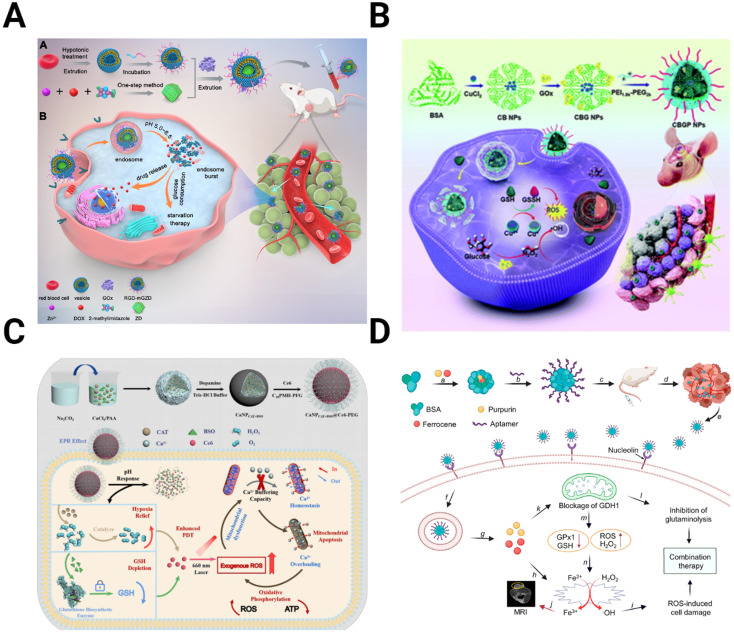
Schematics depicting the preparation and mechanism for NPs targeting metabolic alterations in the TME. (A) ZIF-8 MOFs modified with the red blood cell membrane to deliver GO_*x*_ and DOX for starvation chemotherapy [reproduced from ref. 169 with permission from Elsevier, ©2022]. (B) BSA NPs containing CuO coated with GOx and PEG_2k_-PEI_1.8k_ for targeted Fenton-like reaction and starvation therapy [reproduced from ref. 170 with permission from Royal Society of Chemistry, ©2022]. (C) CaCO_3_ NPs loaded with catalase, Ce6, and buthionine sulfoximine, designed to overwhelm calcium buffering capacity to inhibit ATP production [reproduced from ref. 175 with permission from Elsevier, ©2022]. (D) Targeted BSA NPs encapsulate purpurin to inhibit glutaminolysis and ferrocene for ROS-mediated cell damage [reproduced from ref. 177 with permission from American Chemical Society, ©2021].

Therapies targeting metabolism seek to improve treatment by exploiting changes in the energy balance and cell metabolite production. The increased dependence of the TME on glucose due to the Warburg effect can be targeted to hinder the energy cycle of GBM cells. Glucose oxidase (GO_*x*_) converts glucose into gluconic acid and H_2_O_2_ in the presence of O_2_, cutting off the primary energy source of the tumor and leading to increased tumor stress and cell death.^167,168^ The effectiveness of GOx is improved when incorporated into a multimodal NP system. For example, Ke *et al.* designed a red blood cell membrane-cloaked zeolitic imidazolate framework-8 (ZIF-8), a type of metal–organic framework (MOF), for the co-delivery of GO_*x*_ and DOX (Fig. 6A).^169^ As the GO_*x*_ is released from the MOF and increases tumor acidity, the MOF degrades, releasing DOX into the starved TME. *In vivo* studies with U87 tumors show the improved tumor inhibition capability of the NPs compared to any singular component. Qiao *et al.* utilized GO_*x*_-modified bovine serum albumin (BSA) NPs with the cationic polymer PEG_2k_-PEI_1.8k_ as a coating to prolong circulation time (Fig. 6B).^170^ These NPs encapsulated CuO for delivery, combining Fenton-like reactions and starvation therapy for more effective antitumor activity.

Similarly, a fibrin gel containing IDH1 was used for GBM starvation therapy.^171^ After tumor resection, the gel containing Mn-doped calcium phosphate NPs encapsulating GO_*x*_ is sprayed into a surgical defect. These NPs utilized a combination of Fenton-like generation of H_2_O_2_ and glucose starvation therapy. GBM starvation therapy is also achieved by inhibiting the overexpressed glucose transporter Glut1. CD44-targeting gold nanorods conjugated with a Glut1 inhibitor to interfere with the energy balance in GBM cells.^172^ By inhibiting Glut1, ATP levels are reduced, decreasing the expression of heat shock proteins (HSP), which have increased expression in cancer and play an essential role in maintaining thermoresistance.^173^

An alternative strategy for altering cancer cell metabolism is directly targeting ATP synthesis. In addition to powering function and proliferation of GBM cells, intracellular ATP concentrations drive drug resistance.^174^ Zhu *et al.* overloaded the GBM mitochondria with Ca^2+^ to induce cell apoptosis (Fig. 6C).^175^ The authors fabricated CaCO_3_ NPs loaded with catalase, Ce6, and buthionine sulfoximine (BSO) to combine oxygen and GSH depletion with calcium overloading. While Ca^2+^-related toxicity would be limited on its own, ROS production from GSH and O_2_ depletion reduce the buffering capacity of the mitochondria, allowing them to be more easily overwhelmed and resulting in greater therapy efficacy.

Another target within the metabolism besides glucose is glutaminolysis. Like glycolysis, glutaminolysis is upregulated in the TME, driving the TCA cycle and the production of antioxidants such as GSH.^176^ Xu *et al.* take advantage of this by encapsulating purpurin, the inhibitor of glutamate dehydrogenase 1 (GDH1), an essential enzyme in glutaminolysis, in a BSA NP (Fig. 6D).^177^ When combined with the H_2_O_2_-producing ferrocene, purpurin disrupts cell metabolism and inhibits cancer growth.

### 4.5 Extracellular matrix

The last area to be discussed is the application of NP systems to modify the ECM. These therapies target components of the ECM with the primary goal of inhibiting those that increase tumor aggressiveness.

The first group of ECM-targeting therapies seeks to reduce the density of the ECM within the TME. One method of accomplishing this task is to utilize a proteolytic enzyme to degrade the matrix. Zinger *et al.* designed a liposome system encapsulating collagenase to enhance the transport of a drug into the dense tumor core of pancreatic ductal carcinoma.^178^ When administered intravenously, these liposomes decreased the fibrotic tissue mass to approximately 5%, meaning the dense collagen network within the TME had been deconstructed. Collagen depletion should be treated cautiously, as it can induce the release of pro-inflammatory cytokines and growth factors that promote tumorigenesis.^179^ Losartan, an angiotensin inhibitor, reduces the synthesis of HA and collagen, reducing stress on the tumor and improving perfusion.^180^ Zhao *et al.* combined losartan with anti-PD1 therapy to enhance the efficacy of chemo-immunotherapy for triple-negative breast cancer.^181^ Using liposomes to encapsulate DOX, losartan, and α-PD1, the authors could normalize the TME, reducing stromal density, hypoxia, and immunosuppression. The components within the NP were able to reduce tumor size more than compared to only one or two drugs. Grabowska *et al.* chose TN-C as their target for inhibition, utilizing double-stranded RNA (dsRNA).^182^ The dsRNA was encapsulated in a magnetite NP coated with the cationic polymer polyethyleneimine (PEI) for simultaneous drug delivery and MRI imaging. By inhibiting TN-C, the authors reduced the migration and proliferation of GBM cells.

The final strategy is to inhibit MMPs to reduce migration and invasiveness. The most popular drug inhibiting MMP-2 is chlorotoxin (CTX), a peptide derived from scorpion venom.^183^ Agarwal *et al.* incorporated CTX onto a morusin-loaded PLGA NP for GBM treatment.^184^ Combining MMP-2 inhibition through CTX with morusin, which inhibits MMP-2 and MMP-9 in hepatoma SK-Hep1 cells, produced a potent cytotoxic effect *in vitro*.^185^ In addition, morusin stimulates apoptosis by inhibiting the NF-κB and STAT3 pathways and antioxidant scavenging.^185–187^

## 5. Outlook

There is interest in engineering the TME with nanotechnology, leading to a fast-moving landscape of innovations. One area of particular interest is combination therapies, where one or more TME-altering agents are used in concert with a traditional therapy within an NP system. However, there are still areas where TME therapies have not yet been explored for GBM. For example, while acidity is a core component of the TME, no therapies specifically target it. Hypoxia, oxidation state, metabolism, or immunosuppression are interdependent within the TME. While hypoxia reduction inadvertently reduces acidity, assessing their relative impact would advance our understanding of the TME. In addition, while target molecules like CPSG-4 and fibulin-3 are demonstrated to promote cancer aggressiveness, no nanocarrier therapies have been developed to target them. The field is relatively new, with no therapies successfully tested in clinical trials, but it is rapidly expanding. As NP-based therapies enter clinical trials in more significant volumes, it will be vital to understand more thoroughly the effects these treatments have through the lens of the TME, the healthy brain parenchyma, and systemic function. For this reason, there is still much to learn before GBM treatment can be improved and the survival time extended.

## Conflicts of interest

There are no conflicts to declare.

## Supplementary Material
